# Effect of siRNA-silencing of *SALL2* gene on growth, migration and invasion of human ovarian carcinoma A2780 cells

**DOI:** 10.1186/s12885-017-3843-y

**Published:** 2017-12-11

**Authors:** Fang Miao, Xueshan Zhang, Yanning Cao, Yue Wang, Xiaoshu Zhang

**Affiliations:** School of Basic Medical Sciences, Binzhou Medical University, 346 Guanhai Road, Yantai, Shandong People’s Republic of China

**Keywords:** SALL2, Ovarian carcinoma, RNA interference, p21, MMP2/9, PI3K/Akt

## Abstract

**Background:**

The role of *Spalt-like* gene-2 (*SALL2*) in tumorigenesis remains incompletely elucidated. This study investigated the effects of SALL2 on human ovarian carcinoma (OC) A2780 cells and the probable mechanism.

**Methods:**

Expression of SALL2 in human OC cell lines were detected by reverse transcription PCR (RT-PCR) and Western blot analysis. A2780 cells were transfected with small-interfering ribonucleic acid (siRNA) to silence SALL2. SALL2 expression was detected by RT-PCR, Western blot analysis and immunofluorescence assay. Cell proliferation was measured by CCK-8 assay and flow cytometry (FCM). Apoptosis was measured by FCM. Cell migration was detected by real-time cell analysis. Cell invasion was detected by transwell assay. mRNA expression of p21 was detected by quantitative real-time PCR. Western blot analysis was used to determine the expression of matrix metalloproteinase (MMP)2, MMP9, protein kinase B (PKB, also called Akt), and phosphorylated-Akt (p-Akt).

**Results:**

SALL2 was expressed in six OC cell lines, and the expression was the highest in A2780 cells. Compared with that in the Scramble group, SALL2 expression in A2780 was downregulated after transfection with siRNA-2 and siRNA-3 for 48 h. Compared with that in the Scramble group, proliferation of A2780 cells in the siRNA-2 group increased after transfection for 24, 48 and 72 h. In the siRNA-2 group, the proportion of A2780 cells decreased in the G0/G1 phase, and cell apoptosis decreased after transfection for 48 h. Compared with that in the Scramble group, the cell migration and invasion abilities of A2780 cells increased. Compared with that in the Scramble group, p21 mRNA expression in A2780 cells decreased after transfection with siRNA2. When SALL2 was silenced, the expression of MMP2/9 and p-Akt in A2780 cells increased. Furthermore, the PI3K inhibitor LY294002 could effectively reversed SALL2 siRNA-induced phosphorylation of Akt, migration and invasion of A2780 cells.

**Conclusion:**

Transient silencing of SALL2 promotes cell proliferation, migration, and invasion, and inhibits apoptosis of A2780 cells. In SALL2 siRNA-silenced cells, p21 expression was decreased. SALL2 knockdown by siRNA induces the migration and invasion of A2780 cells; this phenomenon is possibly associated with the increased expression of MMP2/9 and the activation of the PI3K/Akt signalling pathway.

**Electronic supplementary material:**

The online version of this article (10.1186/s12885-017-3843-y) contains supplementary material, which is available to authorized users.

## Background

Ovarian carcinoma (OC) is one of the most common causes of postmenopausal cancer mortality worldwide, and epithelial OC (EOC) accounts for most of the histological types [1]. EOC is the second deadly gynecologic cancer and is the fifth leading cause of cancer-related deaths in women, with 22,440 new cases and 14,080 deaths estimated worldwide in 2017 [2]. Despite considerable recent progress in the surgical treatment of EOC, the overall and progression-free survival of EOC remain poor primarily because the initial diagnosis is made at the advanced stages of the cancer, particularly in developing countries [3]. In the past two decades, several new drugs have become available for the treatment of patients with OC. However, the effectiveness of a given treatment in a particular individual cannot be easily determined because objective measures that define efficacy are not available [4]. These factors emphasise the need to identify biomarkers that can facilitate the monitoring of diagnosis, treatment, and prognosis of OC [5].

The *Spalt-like* gene-2 (*SALL2*) is a GC box-binding protein, which contains a DNA-binding sequence GGG(T⁄C)GGG [6]. SALL2 is a putative tumour suppressor and an inactivation target of a polyoma tumour antigen [7, 8]. Moreover, SALL2 plays a major role by increasing its expression in colorectal carcinoma tissues [9]. SALL2 is possibly an early tumour marker for gastric carcinomas [10] and acts as a suppressor through hypermethylation of the SALL2 P2 promoter in OC [11]. Inhibition of tumour growth in SCID mice was caused by the restoration of SALL2 expression in OC cells [12]. *SALL2* is located on chromosome 14q12.1–13, a region associated with the loss of heterozygosity in 25% of bladder cancers and 49% of OCs [13]. Although data imply that SALL2 contributes to the growth of normal human epithelial ovarian cells, the regulatory mechanisms underlying the involvement of SALL2 in tumour growth and metastasis is not fully clear.

In this study, we silenced SALL2 in OC cells by using a siRNA to investigate the role and mechanism of SALL2 in the tumorigenesis in OC. We elucidated the effects of SALL2 knockdown on OC cell growth, migration, and invasion as well as on the potential migration and invasion molecular mechanisms that accompany the enhanced expression of MMP2 and MMP9.

## Methods

### Cell lines and culture conditions

Seven OC cell lines (COC1, HO8910, OVCAR-3, HEY, CAOV3, A2780, and SKOV3; the catalogue numbers of these cell lines are 3111C0001CCC000368™, 3131C0001000700024™, 3131C0001000700108™, 3131C0001000700111™, 3111C0001CCC000339™, 3111C0002000000075™ and 3131C0001000700107™, respectively.) were obtained from Cell Bank of Shanghai Institutes for Biological Sciences (Shanghai, China). COC1 and CAOV3 were maintained in RPMI 1640 medium (Gibco, Carlsbad, CA, USA) containing 10% foetal bovine serum (FBS, Invitrogen, Carlsbad, CA, USA); HO8910 was maintained in Dulbecco’s modified Eagle medium high glucose (DMEM/HG) containing 10% FBS. The other cell lines were maintained in DMEM/F12 containing 10% FBS.

### RNA interference

The cells were divided into three groups: Blank control group (untreated), Scramble group (transfected with nontarget siRNAs), and SALL2 siRNA group (transfected with SALL2 siRNAs). The A2780 cells were transfected with three SALL2 siRNAs, namely siRNA1 duplexes (sense: 5′-CCAGCAGUGGCUUGCCUUAUGGUAU-3′; antisense: 3′-GGAAGGAGAUGGACAGUAAUGAGAA-5′), siRNA2 duplexes (sense: 5′-AUACCAUAAGGCAAGCCACUGCUGG-3; antisense: 3′-CAACAACUCUUCGGCCUCCUCUGAA-5′), and siRNA3 duplexes (sense: 5′-UUCUCAUUACUFUCCAUCUCCUCCUCCC-3; antisense: 3′-UUCAGAGGAGGCCGAAGAGUUGUUG-5′). Lipofectamine™ RNAiMAX (Invitrogen, Carlsbad, CA, USA) (9 μl) was added to Opti-MEM (250 μl) and mixed for 5 min. Each siRNA (Invitrogen, Carlsbad, CA, USA) (3 μl) and Opti-MEM (250 μl) were mixed. The diluted Lipofectamine and siRNA were mixed for 15 min. The reagents were added into six-well plates, in which A2780 cells were seeded (5 × 10^5^ cells/well) for 24 h. The cells in the Scramble group were treated with Stealth™ RNAi Negative Control Duplex (Invitrogen). The positive control cells were treated with BLOCK-iTTM Alexa Fluor® Red Fluorescent Oligo. The transiently transfected cells were assayed through quantitative real-time PCR (qRT-PCR) and Western blot analysis after transfection for 48 h.

### Confocal laser scanning microscopy (CLSM) analysis

The transfected A2780 cells at a density of 1 × 10^6^ cells/mL were cultured on 35-mm glass-based culture dishes containing DMEM with 10% FBS at 37 °C for 24 h under 5% CO_2_. The cells were fixed and permeabilized, followed by staining overnight with mouse anti-Human SALL2 (1:50) mAb in a humidified box at 4 °C. The secondary CY5-conjugated goat anti-mouse antibody (1:100) was subsequently added and incubated for 1 h at room temperature. The cells were washed in cold PBS two times for 3 min and then analysed through CLSM (Olympus, IX71, Tokyo, Japan). The nuclei of the cells were stained with Hoechst 33,258 (Amresco, USA). Isotype controls (Invitrogen, Carlsbad, CA, USA)were used in each experiment.

### Cell proliferation assay

At 48 h post transfection of the A2780 cells with siRNA, 4 × 10^3^ cells/mL were introduced into a 96-well plate at 100 μl/well. The cells were incubated at 37 °C under 5% CO_2_. They were subsequently incubated for an additional 2 h with 10 μl CCK-8 (Dojindo, Kumamoto, Japan) for 24, 48, and 72 h. The absorbance at 450 nm was measured using a microplate reader (Tecan M200 PRO, Switzerland). Cell proliferation ability was determined as follows: cell proliferation ability = AV (Absorbance value)/0 h AV.

### Cell apoptosis analysis

At 48 h post transfection of the A2780 cells with siRNA, 1 × 10^5^ cells/mL were introduced into a 24-well plate at 500 μl/well. The cells were cultured at 37 °C for 24 h under 5% CO_2_ according to the instruction manual of the Annexin V-FITC/propidium iodide (PI) Cell Apoptosis Detection Kit (KeyGEN BioTECH, Nanjing, China). The cells were subsequently treated with 0.5 μg/ml cisplatin (Hansoh Pharmaceutical Co., Ltd., Lianyungang, China) for 18 h, and then digested with 0.25% trypsin (without EDTA), washed with PBS, centrifuged at 2000 rpm for 5 min, and collected. The collected cells were suspended in 500 μl of binding buffer to which 5 μl of Annexin V-FITC and 5 μl of PI were added. The mixture was incubated in the dark for 15 min at room temperature and analysed through flow cytometry (FCM, FACS Aria III, Becton Dickinson, USA).

### Cell cycle assay

At 48 h post transfection of the A2780 cells with siRNA, 2.5 × 10^5^ cells/mL were introduced into a 6-well plate at 2 ml/well. All adherent and floating cells were harvested, fixed gently in 70% ethanol overnight at 4 °C, and resuspended in 500 μl of PBS containing 25 μl of PI (20×) and 100 μl of RNase A (50×). After incubation at 37 °C in the dark for 30 min, the cells were analysed by FCM. Data were analysed using the Cell Quest software (BD Biosciences, San Jose, CA, USA).

### RT-CIM migration assay

An xCELLigence Real-Time Cell Analyzer (RTCA) DP (ACEA Biosciences, San Diego, CA, USA) was used in this study. Cell-culture media (100 μl) was added in the lower chamber of the system at room temperature. A CIM-plate 16 was connected to the system. The cell-culture incubator was examined to ensure appropriate electrical contacts, and the background impedance was measured. At 48 h post transfection with siRNA, the cells were resuspended in a cell-culture medium, and the cell density was adjusted to 10^5^ cells/well. Complete medium (165 μl) and serum-free medium (30 μl) were added in the upper and lower chambers, respectively. After 30-min incubation at room temperature, CIM-plate 16 was placed in a cell-culture incubator. Cell migration was monitored every hour for 76 h by using the incorporated sensor electrode arrays of the CIM-plate 16. Electrical impedance was measured using the RTCA-integrated software of the xCELLigence system as a dimensionless parameter termed CI.

### Transwell assay

Cell invasion was assessed using 24-well inserts (BD Biosciences Cat. No. 354480) with 8-μm pores according to manufacturer instructions. In brief, 48 h post transfection with siRNA, 1 × 10^5^ cells were seeded into the upper chamber with (to measure invasion) or without (to measure migration) the matrigel layer and were allowed to invade the lower reservoir containing 20% FBS at 37 °C for 24 h. After 48 h, the noninvading cells in the upper surface of the filters were removed using a cotton swab; the cells were fixed in 4% paraformaldehyde for 30 min and then stained with 0.1% crystal violet for 5 min. The cells in five visual fields that passed through the membrane were counted as invasive cells. All the cells were counted using a microscope at ×200 magnification.

### Real-time fluorescence quantitative PCR (qPCR)

The relative expression levels of SALL2 and p21 were measured through qRT-PCR. The total RNA was extracted using the Trizol reagent (Takara, Dalian, China) and then reverse-transcribed into cDNA by using a Revert Aid First Strand cDNA Synthesis Kit (Fermentas, Canada). The forward and reverse primers used for SALL2 DNA amplification were 5′-CATCCTCAGCCTCTTCTGGA-3′ and 5′-TGCAGAGTGACAGCATTGG-3′, respectively; the forward and reverse primers used for p21 DNA amplification were 5′-CAGGTCCACATGGTCTTCCT-3′ and 5′-TGCCCAAGCTCTACCTTCC-3′. The housekeeping gene GAPDH was used as an internal control, and the primers used for GAPDH were 5′-GCGGGGCTCTCCAGAACATCAT-3′ and 5′-CCAGCCCCAGCGTCAAAGGTG-3′. The qPCR reaction system consisted of 10 μl of FastStart Universal SYBR Green Master Kit (Roche Diagnostics, Basel, Switzerland), 0.5 μl of forward and reverse primers (2.5 μM), respectively, 1 μl of cDNA, and 8 μl of ddH_2_O. Probe amplification was performed as follows: 15 s at 95 °C; 45 cycles at 95 °C for 5 s and at 60 °C for 30 s. The relative mRNA expression levels of SALL2 and p21 were normalised against GAPDH by using the comparative △△Ct method. The relative fold change of gene was calculated using the 2^−△△Ct^ formula.

### Western blot analysis

At 48 h after transfection, the cells were collected and lysed using the RIPA lysis buffer (Beyotime, China). A BCA Kit (Pierce, Rockford, IL, USA) was used to estimate the protein concentration. Protein samples (30 μg/lane) were diluted in 5× SDS-PAGE sample-loading buffer and electrophoretically separated on a 10% SDS-PAGE gel. After the proteins were transferred onto polyvinylidene difluoride membranes (Millipore, Bedford, MA, USA), the membranes were blocked with 5% nonfat skimmed milk for 2 h at room temperature and then incubated overnight with primary antibodies at 4 °C. After being washed for 5 min three times, the membranes were incubated with suitable secondary antibodies (1:6000; horseradish peroxidase-labelled) for 1 h at room temperature. Signals were detected using ECL reagents (Pierce, USA). ImageJ software was used to measure the grey value of molecular bands. The following antibodies were used: mouse anti-human SALL2 monoclonal antibody (mAb) (Santa Cruz, CA, USA), rabbit anti-human MMP-2 mAb (San Eagle BioTECH, Wuhan, China), rabbit anti-human MMP-9 mAb (San Eagle BioTECH, Wuhan, China), rabbit anti-human Akt mAb (San Eagle BioTECH, Wuhan, China), rabbit anti-human p-Akt mAb (Bioworld TECH, Nanjing, China), mouse anti-human GAPDH mAb (San Eagle BioTECH, Wuhan, China), rabbit anti-human GAPDH polyoclonal antibody (Goodhere BioTECH, Hangzhou, China) HRP-conjugated goat anti-mouse antibody, and HRP-conjugated goat anti-rabbit antibody (both from Zhongshanjinqiao BioTECH, Beijing, China).

### Inhibitor assay

LY294002 (Cell Signalling Technology, Boston, USA), a PI3K inhibitor, was prepared in DMSO from a stock concentration of 50 mM. The A2780 cells were detached by trypsinization and washed two times with PBS. At 48 h post transfection with siRNA, the transfected cells were cultured in 6-well plates (5 × 10^5^ cells/well) for 24 h and then incubated with 50 μM of DMSO or LY294002 for 30 min. The cell pellets were subsequently collected for Western blot analysis. The following antibodies were used: mouse anti-human Akt mAb (San Eagle BioTECH, Wuhan, China) and rabbit anti-human p-Akt mAb (San Eagle BioTECH, Wuhan, China). For the transwell assay, transfected cells were cultured overnight in 24-well plates (1 × 10^5^ cells/well). Either DMSO (50 μM) or LY294002 (50 μM) was added to the cells for 30 min and then the cells were collected for the transwell experiment.

### Statistical analysis

All data were analysed using SPSS version 17.0 for Windows. Continuous variables were expressed as means ± standard deviations. Differences between the Scramble and SALL2 siRNA groups were assessed using the Student’s t-test; comparisons involving three or more groups were performed using one-way ANOVA. At least three repetitions (*n* = 3) were performed on the data in each group. *P* < 0.05 was considered statistically significant.

## Results

### SALL2 is expressed in human OC cell lines

**Fig. 1:**
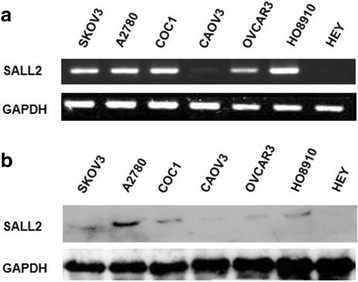
SALL2 expression in human ovarian carcinoma (OC) cells. (**a**) SALL2 expression was measured through RT-PCR. (**b**) Protein expression of SALL2 was investigated through Western blot analysis. The SALL2 expression was high in the A2780 cells than in the six other OC cells lines

RT-PCR and Western blot analysis were used to determine SALL2 expression in seven human OC cell lines (A2780, COC1, HO8910, SKOV3, CAOV3, HEY, and OVCAR3). The results indicated that gene and protein expression of SALL2 were detected in the SKOV3, A2780, COC1, CAOV3, OVCAR3, and HO8910 cells. The protein expression levels of SALL2 were the highest in the A2780 cells; hence, we selected the A2780 cells for subsequent experiments (Fig. 1).

### SALL2 in A2780 cells successfully transient down-regulation

**Fig. 2:**
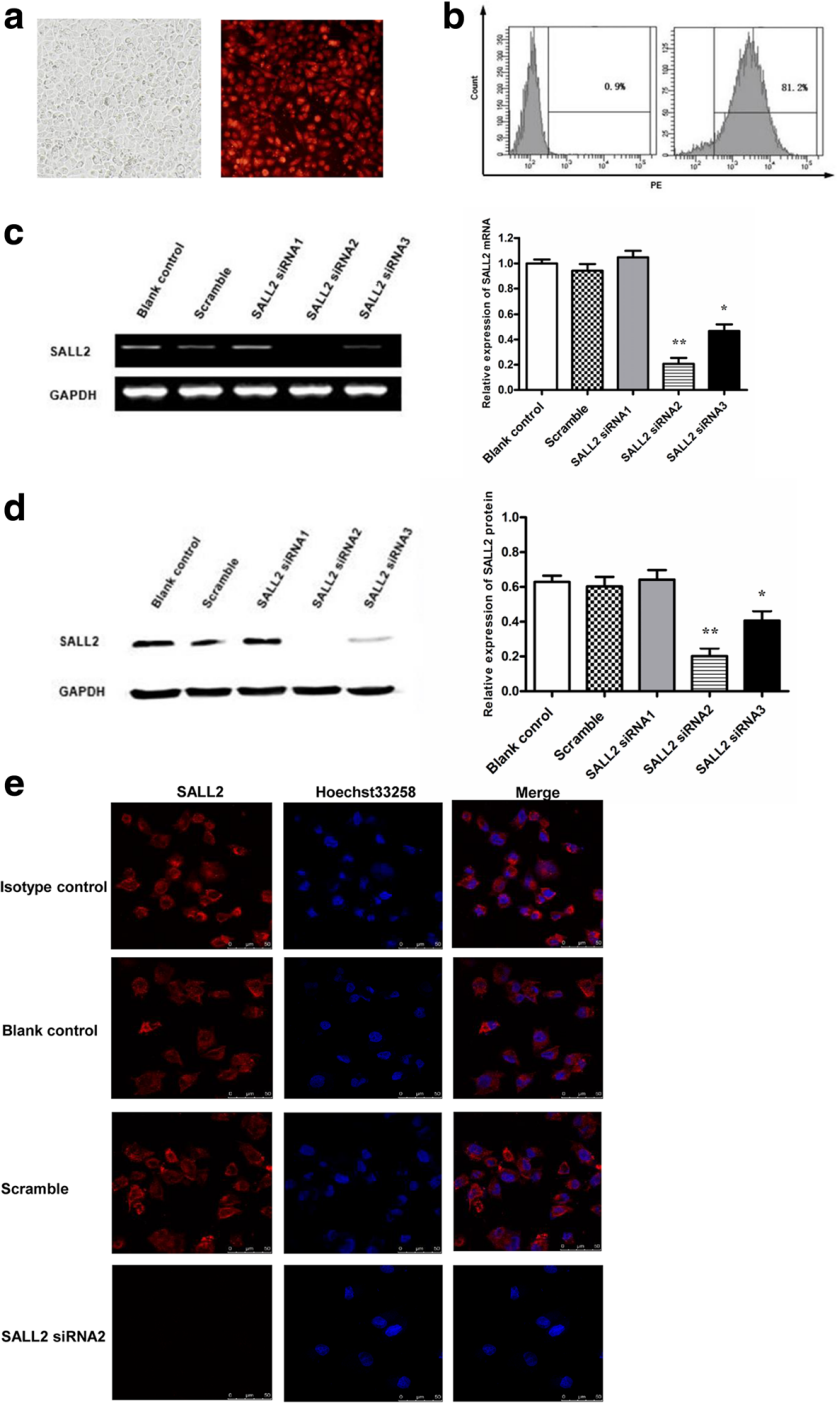
SALL2 downregulation after 48 h of siRNA treatment. (**a**) Fluorescence photography and (**b**) FCM were used to determine the transfection of positive control siRNA into A2780 cells. (**c**) The efficiency of SALL2 silencing by siRNA in the A2780 cells. SALL2 expression was detected by qRT-PCR. (**d**) Western blot analysis indicated SALL2 silencing at the protein level. (*P < 0.05, **P < 0.01, *n* = 3). (**e**) Confocal microscopic analyses confirmed the intracellular SALL2 expression in the Blank control, Scramble, and SALL2 siRNA groups. The cells in each group were stained with purified mouse anti-human SALL2 (1:50) mAb and then with the secondary CY5-conjugated goat anti-mouse antibody (red). The cell nuclei was stained using Hoechst 33,258 (blue). Scale bars, 10 μm

In the A2780 cells, siRNAs were used to downregulate SALL2 expression. The results indicated that the transfection efficiency of the positive control was 81.2%, indicating that SALL2 siRNAs were successfully transfected into the A2780 cells (Fig. 2a and b). SALL2 expression was significantly lower in the siRNA2 group than in the siRNA1, siRNA3, Scramble, and Blank control groups (*P* < 0.01). Moreover, SALL2 expression was lower in the siRNA3 group than in the siRNA1, Scramble, and Blank control groups (*P* < 0.05), and SALL2 expression did not significantly differ among the siRNA1, Scramble, and Blank control groups. Because siRNA2 efficiently silenced SALL2, it was selected for subsequent silencing experiments (Fig. 2c and d). SALL2 siRNAs transfection regulated SALL2 mRNA levels at 24 h and 72 h. (Additional file 1: Figure S1). SALL2 expression in the A2780 cells transfected with siRNA was further confirmed by CLSM. The results indicated that in the Blank control and Scramble group, SALL2 was detected both in the cytoplasm and nucleus. By contrast, SALL2 was not detected in the cytoplasm or nucleus of the cells in the siRNA2 group (Fig. 2e).

### SALL2 silencing promotes growth of A2780 cells

**Fig. 3:**
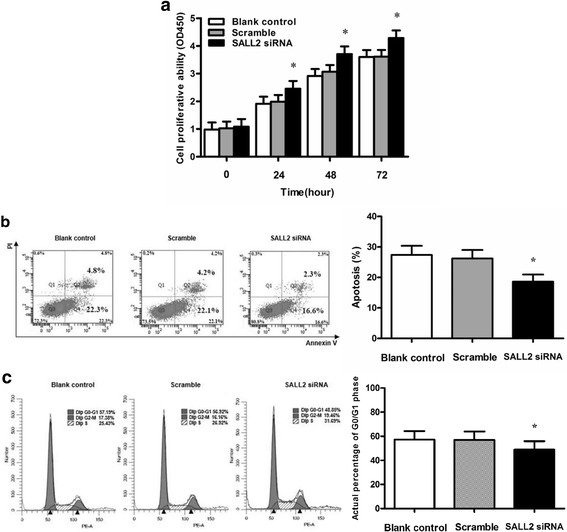
SALL2 silencing by using siRNA treatment for 48 h, and the effect of SALL2 expression on the growth abilities of the A2780 cells. (**a**) CCK-8 was used to determine the proliferation of A2780 cells. Cell proliferation ability = AV (Absorbance value)/0 h AV. (**b**) Cell apoptosis was detected through FCM with Annexin V-PI staining. (**c**) The cell cycle was analysed using FCM. SALL2 silencing by siRNA promoted cell proliferation and cell cycle progression and reduced the apoptotic rate. (*P < 0.05, siRNA vs Blank control or Scramble; *n* = 3)

To determine the role of SALL2 in the growth of OC cells, we analysed the proliferative ability, apoptosis extent, and cell cycle status of the A2780 cells transfected with SALL2 siRNA. At 24, 48, and 72 h, the A2780 cells transfected with SALL2 siRNA exhibited higher levels of cell viability than did the cells in the Scramble group (*P* < 0.05) (Fig. 3a). The A2780 cells transfected with SALL2 siRNA for 48 h exhibited a lower extent of apoptosis than did the cells of the Scramble group (*P* < 0.05) (Fig. 3b; 22.3% early apoptotic cells and 4.5% late apoptotic cells in Scramble group vs. 16.2% and 2.6% in A2780 cells at 48 h after siRNA infection). The number of A2780 cells transfected with SALL2 siRNA in the G0/G1 phase was lower than that of the cells in the Scramble group (*P* < 0.05) for 48 h (Fig. 3c; 56.94% G0/G1 phase cells in Scramble group vs. 48.87% in A2780 cells at 48 h after siRNA infection). Despite these disparities, no significant differences were observed between the Scramble and Blank control groups in terms of proliferation, cell cycle, and apoptosis extent (*P* > 0.05). These results indicated that SALL2 downregulation promoted tumour cell proliferation and cell cycle progression and reduced cell apoptosis.

### SALL2 silencing can promote migration and invasion of A2780 cells

**Fig. 4:**
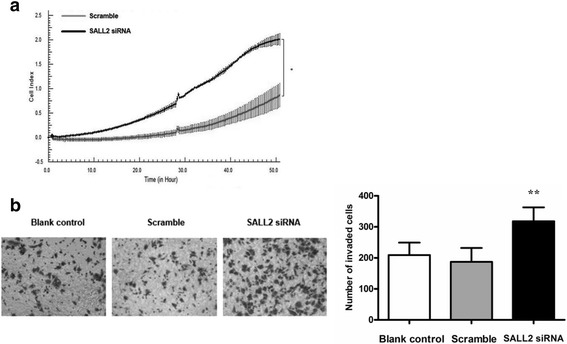
Effect of SALL2 silencing, after siRNA treatment for 48 h, on the migratory and invasive ability of A2780 cells. (**a**) An RT-CIM migration assay was performed to compare the motility of the cells in Scramble and SALL2 siRNA groups. (*P < 0.05; Student’s t-test). The cell index is the number of cells that can migrate the CIM plate. (**b**) The invasive ability of A2780 cells was evaluated using the transwell assay. (*P < 0.05; siRNA vs. Blank control or Scramble; *n* = 3). Photomicrographs are shown at 200× magnification (stained with crystal violet)

Migration of cancer cells is one of the key factors responsible for cancer metastasis [14]. The results of the RT-CIM migration assay indicated that the cell index significantly increased in cells transfected with SALL2 siRNA (*P* < 0.05) compared with the cells in the Scramble group (Fig. 4a). We performed the transwell assay to investigate the invasion capacity of the transfected A2780 cells. The number of invasive cells was significantly higher in the SALL2 siRNA group (*P* < 0.05) compared with that in the Scramble group (Fig. 4b). No significant differences were observed between the Scramble and Blank control groups (*P* > 0.05). These results indicate that SALL2 downregulation promotes tumour cell migration and invasion, resulting in OC cell migration and invasion.

### p21 is essential in SALL2 downregulation to promote growth of A2780 cells

**Fig. 5:**
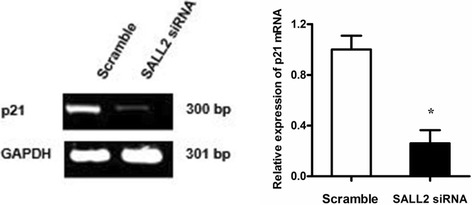
SALL2 silencing after siRNA treatment for 48 h promoted the growth of the A2780 cells by downregulating p21 expression. mRNA expression of p21 in the A2780 cells after SALL2 silencing was detected through qRT-PCR

To investigate the mechanism of the effects of SALL2 expression on growth of the A2780 cells, we detected p21 by using qRT-PCR. The results revealed that the mRNA expression of p21 was significantly lower in the SALL2 siRNA group than in the Scramble group (*P* < 0.05), thereby indicating that SALL2 silencing downregulates p21 mRNA expression (Fig. 5). Considering that *p21* gene is the downstream target of p53, it constitutes the checkpoint of the G1 phase of the cell cycle to reduce the replication and accumulation of damaged DNA; thus, it inhibits the growth of osteosarcoma cells [15]. We speculate that the promotion of A2780 cell growth by SALL2 downregulation is associated with p21.

### MMP2, MMP9, and PI3K/Akt signalling pathways are essential in SALL2 downregulation to promote the migration of and invasion by A2780 cells

**Fig. 6:**
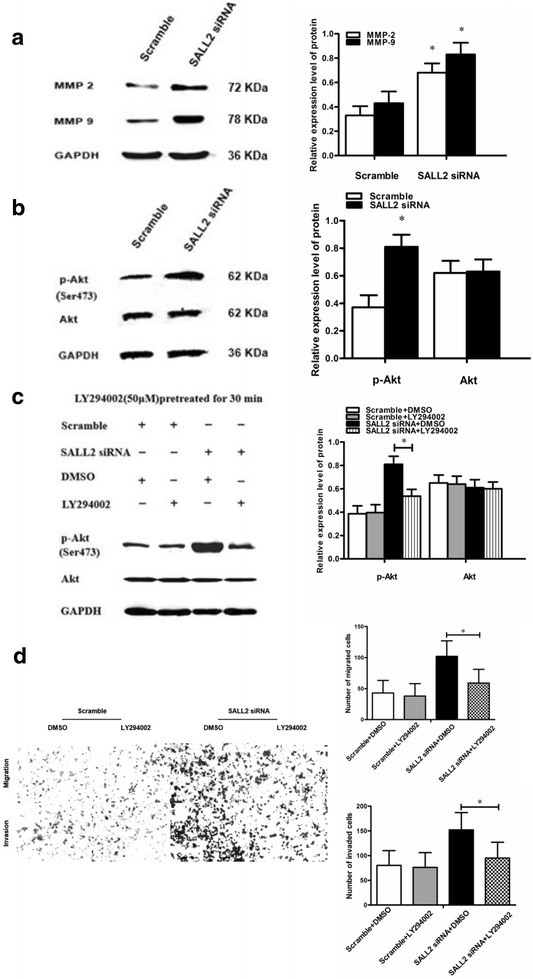
SALL2 silencing for 48 h can promote migratory and invasive behaviour of A2780 cells by upregulating MMP2/9 expression and activating PI3K/Akt. (**a**) The protein expression levels of MMP2 and MMP9 in the A2780 cells after SALL2 silencing was detected by Western blot analysis. (**b**) Protein expression levels of p-Akt and Akt in the A2780 cells after SALL2 silencing were investigated through Western blot analysis. (**c**) Western blot analysis was used to determine the protein expression levels of Akt and p-Akt in the A2780 cells after transfection with Scrambled siRNA or SALL2 siRNA and subsequently treated with 50 μM DMSO or LY294002 for 30 min. (*P < 0.05; SALL2 siRNA + LY294002 vs. SALL2 siRNA + DMSO; *n* = 3). (**d**) Transwell assay was used to assess the migration of A2780 cells after transfection with Scrambled siRNA or SALL2 siRNA and subsequently treated with 50 μM DMSO or LY294002 for 30 min, respectively. (*P < 0.05; SALL2 siRNA+ LY294002 vs SALL2 siRNA+ DMSO; n = 3). Photomicrographs at 200× magnification (stained with crystal violet)

To identify the mechanism underlying the promotion of the migration of and invasion by A2780 cells through SALL2 downregulation, we investigated the MMP2 and MMP9 proteins through Western blot analysis. The results indicated that protein expression of MMP2 and MMP9 was higher in the SALL2 siRNA group than in the Scramble group (*P* < 0.05), thereby indicating that silencing SALL2 upregulates MMP2 and MMP9 protein expression (Fig. 6A).

To investigate whether the migration of and invasion by A2780 cells are correlated with activation of the PI3K/Akt signalling pathway, we detected the Akt and p-Akt proteins through Western blot analysis. The results indicated that protein expression of p-Akt was higher in the SALL2 siRNA group than in the Scramble group (*P* < 0.05). However, the expression level of Akt did not change considerably (Fig. 6B).

In the SALL2 siRNA group, the cells treated with LY294002 exhibited lower expression levels of p-Akt and a lower number of migrating and invading cells than did the cells treated with DMSO (*P* < 0.05). The total Akt protein levels did not differ between the two treatments (Fig. 6c and d). In the Scramble group, the expression of p-Akt and Akt and migration of and invasion by the A2780 cells were not affected by DMSO or LY294002 treatment (*P* > 0.05).

These results suggest that SALL2 downregulation elevates MMP2 and MMP9 protein expression levels and activates the PI3K/Akt pathway, thereby accelerating migration and invasion of OC cells.

## Discussion

The ontogenesis of EOC is a result of various molecular events such as mutation of *p53*, *KRAS*, *BRAF*, and *ERBB2* genes [16]. The mortality rate for EOC remains extremely high because of late diagnosis of the cancer and the frequent failure of conventional treatment strategies. Therefore, identification of cancer-related molecular markers associated with the prognosis of EOC and development of novel anticancer therapies are fundamental steps in EOC treatment [17].

SALL proteins are zinc finger transcription factors present in *Caenorhabditis elegans*, which harbour only one member of the *SALL* gene family. In vertebrates, the SALL proteins are generally encoded by four genes (*SALL1–4*). Four members of the *SALL* gene family play roles in embryonic development and genetic disorders [18, 19]. Mutations in SALL1 cause Townes–Brocks syndrome, which is associated with several developmental defects [20]. SALL3 homozygous mutant mice exhibit abnormalities in the cranial nerves and die shortly after birth [21]. SALL3 regulates the development of cone photoreceptors, particularly their terminal differentiation [22]. SALL4 is a critical transcription factor for pluripotency in embryonic stem cells [23]. Mutations in SALL4 result in Okihiro syndrome, characterised by defective heart and kidney development [24, 25]. Furthermore, SALL4 upregulation plays crucial roles in carcinogenesis in gliomas and gastric cancers [26, 27]. Many tumours, such as synovial sarcomas and tongue squamous cell carcinomas, exhibit high levels of SALL2 expression [28–30]. Related reports have indicated that high levels of SALL2 are expressed in the normal ovary; however, it is not expressed in several OC-derived cell lines such as OVCAR-3 and OVCA432 [6]. However, in this study, both the gene and protein levels of SALL2 were detected in the A2780 and other five OC cell lines. The protein expression levels of SALL2 was higher in the A2780 cells than in the other cells; thus, we selected the A2780 cells for studying the effects of SALL2 on OC cells.

An understanding of the molecular mechanisms involved in OC formation and progression will enable the development of more effective treatments for OC. Moreover, SALL2 has been recognised and characterised as a quiescence factor and is essential in arresting the growth of human fibroblasts under serum deprivation [31]. Upon serum restoration, SALL2 is rapidly degraded as cells reenter the cell cycle [8]. In this study, siRNA was transfected into the OC cell line A2780 to silence SALL2 gene. The results indicated that SALL2 gene silencing promoted the proliferation of the A2780 cells. In addition, SALL2 downregulation caused cell cycle arrest at the G0/G1 phase.

SALL2 regulates the balance between the antiapoptotic and proapoptotic B lymphocyte tumour-2 (Bcl-2) protein families. Moreover, SALL2 can regulate the expression of the *Bcl-2-associated X* (*BAX*) gene to induce cell apoptosis [12]. SALL2 plays a crucial role in cell apoptosis during the growth of human foreskin fibroblasts [32]. In this study, SALL2 expression was downregulated in the A2780 cells through siRNA transfection. The results indicated that SALL2 gene silencing reduces the extent of apoptosis in the A2780 cells. SALL2 is downregulated in many malignancies, including gastrointestinal tumours, ovarian tumours, and certain types of leukaemia [33]. These findings on the loss of SALL2 expression in some solid tumours suggest that SALL2 functions as a tumour suppressor.

The *p16* tumour suppressor gene is one of the targets of the *SALL2* genes; SALL2 upregulates p16 transcription through a SALL2 responsive element, which bears a SALL2 binding site near the proximal region of p16 promoter [34]. SALL2 also plays a role in neuronal development, thereby affecting neurite outgrowth. In the nerve cells, SALL2 is the transcriptional promoter of p21, which is also a member of cell cycle-dependent kinase inhibitor family, similar to p16 [35]. Furthermore, SALL2 overexpression can upregulate the activity of p21 promoter in human embryonic kidney cells and EOC [6]. Thus, SALL2 regulation is vital for suppression of tumour growth. Our results suggested that SALL2 knockdown downregulated the level of p21 in the A2780 cells, thereby indicating that SALL2 restrains the proliferation and cell cycle progression of the A2780 cells through transcriptional activation of p21. This finding is similar to the report of Zhenghua Wu et al. [34]. The authors reported that SALL2 blocked cell cycle progression by regulating the promoter activity of p16 in another OC cell line, namely SKOV3.

In addition to the effect of SALL2 on tumour growth, we studied the effect of SALL2 on tumour migration and invasion. SALL2 interacts with other transcription factors to participate in cell reprogramming and inhibition of the migration and invasion of malignant glioma cells following ionising radiation [36]. Furthermore, SALL4 is involved in invasion of different tissues by various epithelial cancers, such as colon villous epithelial cancer and prostate cancer [37]. The current study demonstrated that SALL2 silencing in the A2780 cells resulted in high metastatic potential in vitro. This observation suggests that SALL2 inhibits the metastatic potential of the A2780 cells.

A vital reason for the occurrence of migration and invasion is the infiltration of tumour cells into the extracellular matrix (ECM). MMPs play a crucial role in this process. MMPs mainly degrade a variety of ECM components. Degradation of the basement membrane (BM) in the ECM is the key event in tumour invasion. MMP2 and MMP9 are two major factors in the MMP family, which can specifically degrade collagen V, VII, and X in BM. Furthermore, gelatin and elastic fibres and are positively associated with tumour invasion and migration [38, 39]. Studies have indicated that the invasiveness of OC cell lines is correlated with MMP2 and MMP9 expression [40, 41]. This study found that SALL2 silencing upregulated the protein expression levels of MMP2 and MMP9 in the A2780 cells, thereby suggesting that SALL2 silencing participates in the migration of and invasion by the A2780 cells by influencing the expression levels MMP2 and 9.

The PI3K/Akt/mTOR signalling pathway considerably affects diverse cellular processes involving cell cycle progression, metastases, and angiogenesis [42]. Akt activation results in the phosphorylation of its downstream molecules, including mTOR, NF-κB, and GSK-3β, which regulate the impacts of Akt on cell growth and metastases [43]. The activation of the PI3K/Akt pathway may also upregulate the expression levels of MMP2 mRNA and protein and the degradation of various ECM components to promote tumour metastasis [44].

This study suggested that after transfection with SALL2 siRNA, the levels of p-Akt in the A2780 cells significantly increased, whereas the PI3K inhibitor, LY294002, effectively reversed the SALL2 siRNA-induced Akt activation. Additionally, our results indicated that increased cellular motility caused by SALL2 knockdown can be eliminated by LY294002, thereby confirming that PI3K/Akt is a key signalling pathway through which SALL2 regulates the migratory and invasive abilities of OC cells. Taken together, the promotion of cell metastasis after loss of SALL2 is possibly related to ECM degradation and PI3K/Akt signalling pathway activation in OC cells. Previous studies have suggested the role of SALL2 in OC progression. However, elucidating the function and detailed molecular biological mechanisms through which SALL2 suppresses EOC growth, migration, and invasion requires further in vivo and in vitro investigations.

## Conclusion

Our study demonstrated that SALL2 downregulation effectively promoted growth of the OC cell line, A2780, and this effect is likely related to the downregulation of the *p21* gene. SALL2 functions as a tumour suppressor to inhibit A2780 cell migration and invasion. The effect of SALL2 on migration and invasion of the A2780 cells is likely regulated by the activation of the PI3K/Akt pathway and downregulation of MMP2 and MMP9.

## Additional file

Additional file 1: Figure S1. a. Expression of SALL2 mRNA at 24 h post transfection in A2780 cells. b. Downregulation of SALL2 mRNA expression at 72 h post transfection in A2780 cell. (PPTX 140 kb)

## Abbreviations

CCK-8: Cell counting kit-8
DMSO: Dimethyl sulfoxide
FITC: Fluorescein Isothiocyanate
PBS: Phosphate buffer saline
PI: Propidium Iodide
RT-PCR: Real time polymerase chain reaction
SDS-PAGE: Sodium dodecyl sulfate polyacrylamide gel electrophoresis
