# Supplementary figures and images for: Effect of siRNA-silencing of SALL2 gene on growth, migration and invasion of human ovarian carcinoma A2780 cells
Source: BMC Cancer. 2017 Dec 11;17:838. doi: 10.1186/s12885-017-3843-y (PMC5725831; doi:10.1186/s12885-017-3843-y)

## Slide 1
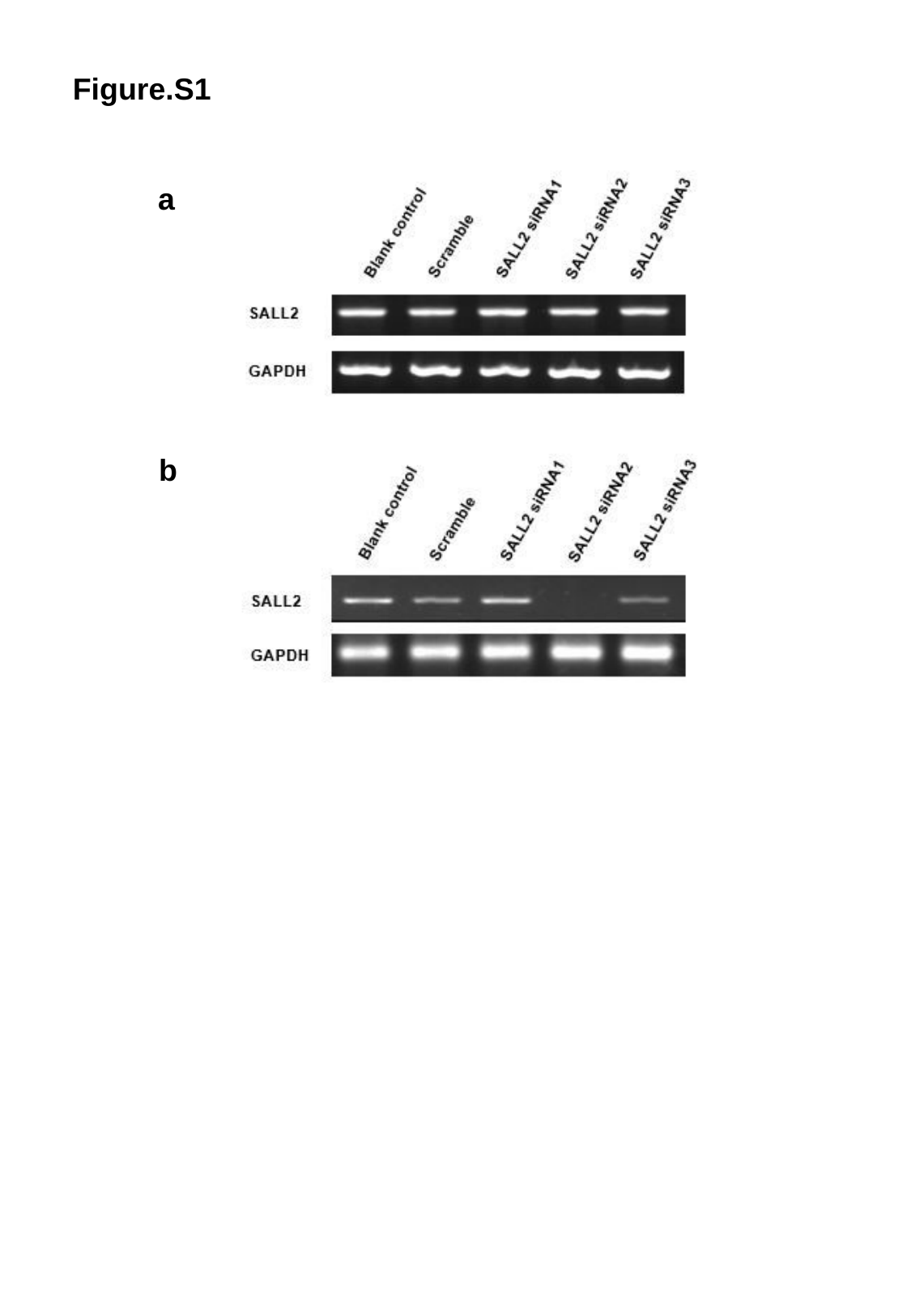

Figure.S1
a
b

Supplement: Additional file 1: Figure S1. — a. Expression of SALL2 mRNA at 24 h post transfection in A2780 cells. b. Downregulation of SALL2 mRNA expression at 72 h post transfection in A2780 cell. (PPTX 140 kb) [file 12885_2017_3843_MOESM1_ESM.pptx]
